# The Domain and Structural Characteristics of Ferroelectric Copolymers Based on Vinylidene Fluoride Copolymer with Tetrafluoroethylene Composition (94/6)

**DOI:** 10.3390/polym16020233

**Published:** 2024-01-15

**Authors:** Valentin V. Kochervinskii, Evgeniya L. Buryanskaya, Alexey S. Osipkov, Dmitriy S. Ryzhenko, Dmitry A. Kiselev, Boris V. Lokshin, Aleksandra I. Zvyagina, Gayane A. Kirakosyan

**Affiliations:** 1 Laboratory of Technologies of Polymer Ferroelectrics, Bauman Moscow State Technical University, 141005 Moscow, Russia; osipkov@bmstu.ru (A.S.O.); dsr@bmstu.ru (D.S.R.); 2 Laboratory of Physics of Oxide Ferroelectrics, Department of Materials Science of Semiconductors and Dielectrics, National University of Science and Technology MISIS, 119049 Moscow, Russia; dm.kiselev@misis.ru; 3 Division of Physical-Chemical Research Methods, A.N. Nesmeyanov Institute of Organoelement Compounds RAS, 119334 Moscow, Russia; bloksh@ineos.ac.ru; 4 A.N. Frumkin Institute of Physical Chemistry and Electrochemistry RAS, 119991 Moscow, Russia; zvyagina.ai@gmail.ru (A.I.Z.); gayakira@mail.ru (G.A.K.); 5 Laboratory of Coordination Chemistry of Alkali and Rare Metals, N.S. Kurnakov Institute of General and Inorganic Chemistry RAS, 119991 Moscow, Russia

**Keywords:** polymers, ferroelectricity, piezoelectricity, conductivity, polarization, domain structure, dipole moment, piezoresponse force microscopy, relaxation, phase transition

## Abstract

This paper presents data on the macroscopic polarization of copolymer films of vinylidene fluoride with tetrafluoroethylene obtained with a modified apparatus assembled according to the Sawyer–Tower Circuit. The kinetics of the polarization process were analyzed taking into consideration the contributions of both bound and quasi-free (impurity) charges. It was shown that an “abnormal” decrease in conductivity was observed in fields near the coercive fields. This could be associated with the appearance of deep traps of the impurity charge carriers formed by the polar planes of β-phase crystals. The conductivity data obtained from the charge and current responses differed. It was concluded that chain segments contributing to polarization with sufficiently low fields were present in the amorphous phase. A comparison showed that the average size of β-phase crystals (crystals of X-ray diffraction reflection width) was almost one order of magnitude lower than the domain size obtained using piezoresponse force microscopy (PFM). The analysis of the fast-stage dielectric response before and after polarization indicated that as the external polarizing field increased in the ferroelectric polymer chains, conformational transitions occurred according to the T_3_GT_3_G^−^ → (-TT-)_n_ и TGTG → (-TT-)_n_ types. This was accompanied by an increase in the effective dipole moment in the amorphous phase chains. The analysis of the IR spectroscopy data obtained in transmission and ATR modes revealed a difference in the conformational states of the chains in the core and surface parts of the film.

## 1. Introduction

In this paper, the details of the structure of copolymers of vinylenefluoride with tetrafluoroethylene (VDF/TFE) are investigated. The copolymers of vinylenefluoride are ferroelectric materials with high multifactorial response. This type of polymers is characterized by high values of pyro- and piezoelectric response and electrostrictive and electrocaloric effects after the action of high voltage fields [1,2,3,4]. It was previously shown that the noted responses significantly depend on the domain structure of the material. Considering that the ferroelectric domains in the material form β-phase crystals [5,6], in this paper, the hypothesis is considered according to which the chains of the amorphous phase that also participate in the formation of domains can be distinguished. This phase includes the chains of rigid amorphous phase [7,8]. The authors of [9] investigated copolymers of VDF in various states using NMR and rotation at a magic angle. It was shown that the polymer contains mobile and immobilized sections of chains (rigid amorphous phase). In this regard, the details of the domain structure of a ferroelectric polymer based on VDF are considered in this paper.

The structural peculiarities of crystalline polymers, e.g., the long-range order along the axis of the macromolecule, are reduced to the fact that the system becomes structurally heterogeneous under the normal conditions of crystallization. As in crystals, the so-called amorphous phase, where there is no long-distance order, is present in the volume. Ferroelectric polymers based on vinylidene fluoride (VDF) belong to this category of the substance condensed state, whose fraction is 0.5 and higher [1,2,3,4]. The noted polymers, along with polyethylene (PE) and isotactic polypropylene (iPP) [10,11], are flexible-chain polymers. In terms of macroscopic properties, this is manifested because the glass transition temperature in VDF copolymers is ~−40 °C. This means that at room temperature (and higher), where various types of sensors made of these materials could be operated, the amorphous phase is in a liquid-like state, where the average relaxation periods in the cooperative segmental mobility are ~100 ns [12]. This circumstance significantly affects the compliance of the considered heterogeneous medium, which significantly changes the mechanism of the electrophysical response in the considered polymers [1,2,3,13,14] in comparison with classical inorganic ferroelectrics.

The noted structural and dynamic characteristics can also appear during domain structure formation in the ferroelectric polymers under consideration. This issue is somewhat widely covered for classical inorganic ferroelectrics, whereas it is less studied in organic polymer ferroelectrics. The study of the domain structure of the material could help to optimize the synthesis of such polymers, improve their electrophysical properties and make it possible to manufacture efficient devices based on them. VDF-based ferroelectric polymers have high piezoresponse and pyroresponse values, present chemical and thermal stability and are biocompatible. This is why they are of significant interest as materials for flexible electronics and biomedicines [12].

This paper presents certain experimental data supporting the hypothesis that structural and dynamic characteristics are related to the domain structure of ferroelectric polymers. The analysis of the structural characteristics and piezoresponse force microscopy (PFM) data shows that the average domain size is an order of magnitude higher than the polar crystal size. This means that the formed domain includes not only crystals of polar modification but also sections of amorphous phase circuits.

## 2. Materials and Methods

### 2.1. Nuclear Magnetic Resonance (NMR)

The vinylidene fluoride copolymer with tetrafluoroethylene P (VDF-TFE) was investigated. The microstructure of its chains was studied with the ^19^F NMR method, employing the ^19^F type of fluorine isotope with a number of protons equal to 19. The spectra of solutions in acetone-*d*_6_ were recorded with H-decoupling at 303 K using the Bruker Avance II spectrometer (Bruker Corporation, Karlsruhe, Germany) operating at the fluorine frequency of 282.48 MHz. The ^19^F NMR chemical shifts were referenced externally to CFCl_3_ (0 ppm).

### 2.2. X-ray Diffraction

X-ray diffraction measurements were performed using the Bruker D8 Advance device equipped with automatic slits (*λ* = 0.1542 nm) and the position-sensitive LynxEye detector (aperture angle of 3°). Assuming the spherical shape of the crystal, the *l_hkl_* crystallite size in the direction normal to the *hkl* plane was determined using the Debye–Scherrer equation:

(1) $$l_{c}=\frac{0.9k\lambda }{\cos\theta \sqrt{\beta^{2}-\beta_{et}^{2}}},$$

where *k* is the diffraction order; *λ* is the wavelength; *β* and *β_et_* are the measured and the etalon peak full widths at half maximum and *θ* is the Bragg angle (diffraction angle).

### 2.3. Fourier-Transform Infrared Spectroscopy

Attenuated total reflectance (ATR) spectra were obtained using the VERTEX 70 (Bruker Corporation, Karlsruhe, Germany) IR Fourier spectrometer in the range of 4000–400 cm^−1^ and the PikeGlady ATR single reflection attachment (PIKE Technologies, Madison, WI, USA) with the diamond working element. The spectra were corrected with a program included in the OPUS 7.0 software to estimate the wavelength dependence of the radiation penetration depth into the sample. Surface polymer layer (0.5–2 μm thick) was scanned. Absorption spectra were measured using the TENZOR 37 IR Fourier spectrometer (Bruker Corporation, Karlsruhe, Germany).

### 2.4. Scanning Probe Microscopy

Topography and Kelvin probe force microscopy (KPFM) of the polymer samples were performed with the Ntegra Prima (NT-MDT SI, Zelenograd, Russia) scanning nano-laboratory using the NSG10/Pt (Tipsnano, Tallinn, Estonia) platinum conductive probe with the spring constant of 12 N/m. For the KPFM measurements, the surface topography was first scanned in the tapping mode and then the 1 V AC voltage was applied on the probe near its resonance frequency (∼180 kHz) to measure the sample surface potential distribution through the DC voltage feedback loop. The scan frequency was set to 0.5 Hz, and the scan rise height was approximately 50 nm. Topography and KPFM images were processed using the Gwyddion 2.6 software.

## 3. Results and Discussion

**Figure 1:**
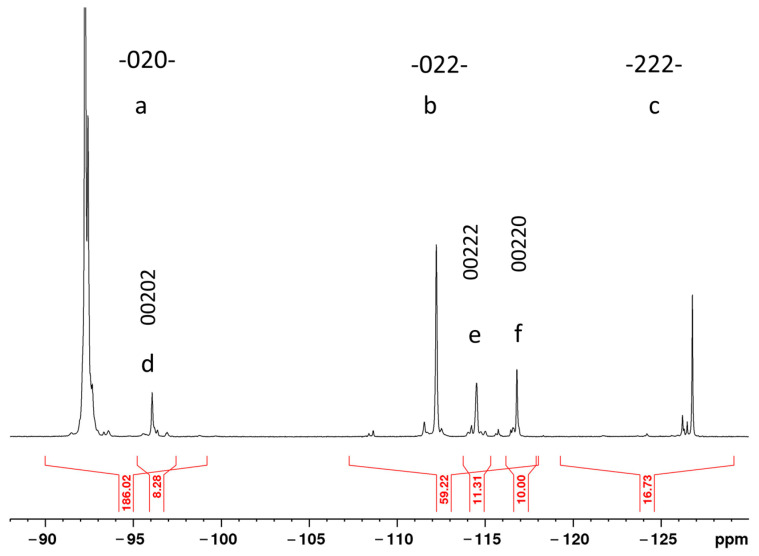
^19^F{^1^H} NMR spectrum of the copolymer film in acetone-d6 with integrated intensities of the resonances required to calculate the composition of the copolymer.

According to the NMR spectrum for ^19^F (Figure 1), the following conclusions could be made.

VF_2_ can attach to the growing chain in two ways, either with the forward directional sense 02, or the reverse sense 20 (the notations 0 and 2 represent the number of fluorine atoms attached to a particular carbon in the polymer chain, i.e., CH_2_ and CF_2_ groups, respectively [15]). The direct attachment mode leads to the usual head-to-tail linkage and produces 020202 sequences. In contrast, the reverse addition mode gives rise to the less probable head-to-head linkage 0220. Copolymers of VF_2_ with F_4_E contain also 22 sequences from tetrafluoroethylene, so that copolymerization can be thought of as introducing an additional 22 defects. In addition, 222 (or even longer 2222… sequences at high F4E contents) defects become possible.

The ^19^F NMR spectroscopy is a powerful tool for studying the long-range differences in structural sequences of the fluorinated polymers. The ^19^F NMR spectrum of the copolymer solution in acetone- *d*_6_ (~3%) (Figure 1) is typical for the VF_2_—F_4_E copolymers’ spectra [15]. It contains three groups of resonances corresponding to the central fluorines in 020, 022 and 222 carbon-type sequences. The VF_2_ content in the copolymer was calculated from integrated intensities of the ^19^F NMR resonances in the spectrum using the following formula [15]:

mol % VF_2_ = 200 × (2*a* + *b* + *d* + *e* + *f*)/(4*a* + 3*b* + 2*c* + *d* + *e* + *f*), (2)

where *a* = 020; *b* = 022 +220; *c* = 222; *d* = 00202 + 20200; *e* = 00222 + 22200 and *f* = 00220 + 02200 are the integrated intensities of the groups of signals in the spectrum (Figure 1).

According to these calculations, the copolymer contains 93.6 mol % VF_2_ and 6.4 mol % F_4_E.

As in [15], F and R denote the forward 02 and reverse 20 orientations of the VF_2_ unit, respectively, and the F_4_E unit is denoted by T. Thus, all the monomer sequences comprise certain permutations of the F, R and T units. The VF_2_ units percentage added as R is assumed to be constant and equal to the polyvinylidene difluoride value (ca. 5%).

Provided that copolymerization obeys the first-order Markov statistics (and this was shown for the VF_2_—F_4_E system), it is possible to forecast probabilities of appearance of the certain sequences using conditional probabilities of the monomer attachment to the growing chain. Let us estimate these probabilities using the example of sequences of four monomer units.

*p*(F) = 0.95 × (mol % VF_2_/100) = 0.95 × 0.936 = 0.89 *p*(R) = 0.05 × (mol % VF_2_/100) = 0.05 × 0.936 = 0.047 *p*(T) = 0.064 *p*(FFFF) = *p*(F) × *p*(F|F) × *p*(F|F) × *p*(F|F) = 0.89^4^ = 0.63 [02] *p*(FRFF) = *p*(F) × *p*(F|R) × *p*(R|F) × *p*(F|F) = 0.89 × 0.047 × 0.89 × 0.89 = 0.033 [02,20] *p*(FTFF) = *p*(F) × *p*(F|T) *p*(T|F) × *p*(F|F) = 0.89 × 0.064 × 0.89 × 0.89 = 0.045 [02,22] *p*(FRFR) = *p*(F) × *p*(F|R) × *p*(R|F) × *p*(F|R)= 0.89 × 0.047 × 0.89 × 0.047 = 0.0017 [02,20] *p*(FRTF) = *p*(F) × *p*(F|R) × *p*(R|T) × *p*(T|F) = 0.89 × 0.047 × 0.064 × 0.89 = 0.0024 [02,20,22] *p*(FTFT) = *p*(F) × *p*(F|T) × *p*(T|F) × *p*(F|T) = 0.89 × 0.064 × 0.89 × 0.064 = 0.0032 [02,22] etc.

The estimates show that the sequences of the VF_2_ unit attached in the head-to-tail way are the most probable. Among chain defects, the most abundant (with estimated probabilities of 4.5 and 3.3%) are the sequences containing single T or R units among the F units, resulting in 222, 22 and 00 segments.

It should be noted that not all the resonance signals correspond to only one sequence of the monomers; many signals are indistinguishable in the ^19^F NMR spectra recorded at the heptad level. Therefore, a simple comparison of the experimental integrated signal intensities with the forecasted probabilities of certain sequences is incorrect.

**Figure 2:**
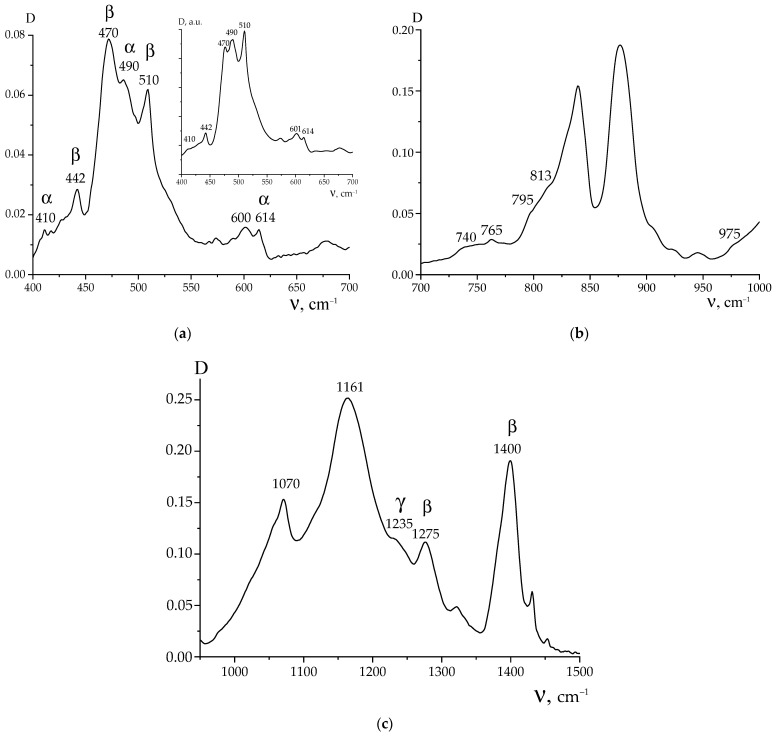
Absorption spectra of the initial copolymer film obtained in the ATR mode in the range from 400 to 1500 cm^−1^ (**a**–**c**); the curve in the inset to (**a**) was obtained in the transmission mode; here, D is optical density.

Phase state of the film under study was characterized by IR spectroscopy and X-ray diffraction at the high angles. Figure 2 shows the absorption spectrum sections, where the conformation-sensitive bands are located.

It follows from the presented spectra that crystallization of the film occurs in the polar β-phase with the planar zigzag (-TT-)_n_ conformation. Indeed, absorption bands at 442, 470, 510,840, 1275 and 1400 cm^−1^ [1,2] are registered in the spectra being characteristic of such conformation. At the same time, other (weaker) bands are observed in the spectra: 410.490, 765, 795 and 813.1235 cm^−1^, which handle isomers in the TGTG- and T_3_GT_3_G- conformations, respectively [1,2,16].

**Figure 3:**
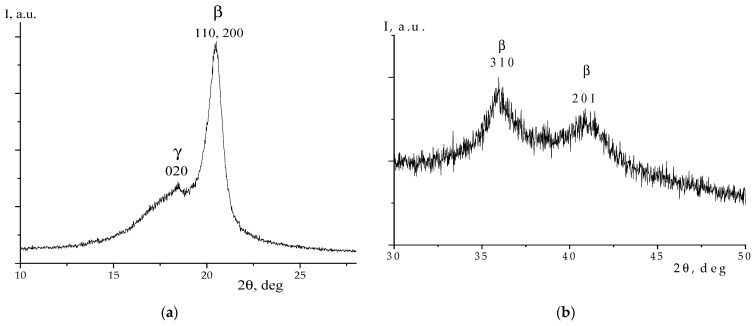
X-ray diffraction from the copolymer film areas of *2θ* from 10 to 28 degrees (**a**) and 30 to 50 degrees (**b**); here, *I* is intensity.

The confirmation of the polar β-phase crystals formation in the film under consideration was also sought by the X-ray diffraction method. The corresponding data are presented in Figure 3. According to the data [1,2,16], crystallization predominantly occurs in the β-phase. The halo of the diffused peak with a maximum near 2θ~18° angles is often referred to as the amorphous phase. In our opinion, it should be associated with the presence of an amorphous phase and a certain amount of the metastable paraelectric phase [16,17]. The TGTG- and T_3_GT_3_G- isomers, which are present in the film (Figure 2), additionally confirm this conclusion, since, according to the data [1,2,3,16], the paraelectric phase is formed by chains in the indicated conformations.

**Figure 4:**
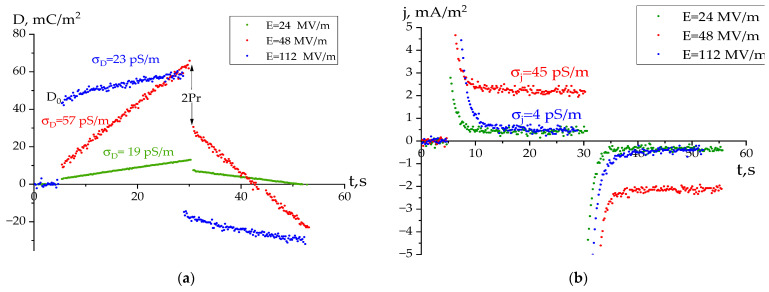
Charge (**a**) and current (**b**) response kinetic curves with the bipolar rectangular pulse of the different intensity field being applied to the copolymer film, *D* is displacement (surface charge density), *j* is the surface current density.

Kinetic curves of the charge response (Figure 4a) to an external field *E* were described by the general equation for a ferroelectric with the conductivity σ, where the contribution to the electric displacement *D* of both bound (third term in Equation (3)) and quasi-free (second term in Equation (3)) charges is taken into consideration.

(3) $$D=\varepsilon_{0}E+\sigma t^{m}E+2P_{r}\left[1-exp\left(-{\left(\frac{t}{\tau_{s}}\right)}^{n}\right)\right],$$

where *t* is the time; *P_r_* is the residual polarization; *τ_s_* is the average switching time of the spontaneous polarization and m and n are the empirical coefficients. In the first approximation, the contribution to the *D-t* curve should be provided by the second term of Equation (3). However, taking into account the complex hierarchical structure of crystallizing polymers, the contribution of the third term to it is not excluded. In this case, the resulting conductivity values were denoted as *σ_D_*. On the other hand, the current response curves (Figure 4b) can be characterized by quasi-stationary values of the current density *j*. They should characterize the conductivity at selected times of the field pulse duration in conditions when a spatial charge is formed and the current is determined mainly by quasi-free carriers. The marked current for the one-dimensional case (the direction of the normal to the surface of the film) can also be calculated by the conductivity *σ_j_* from Equation (4):

*j = σ_j_ E*, (4)

**Figure 5:**
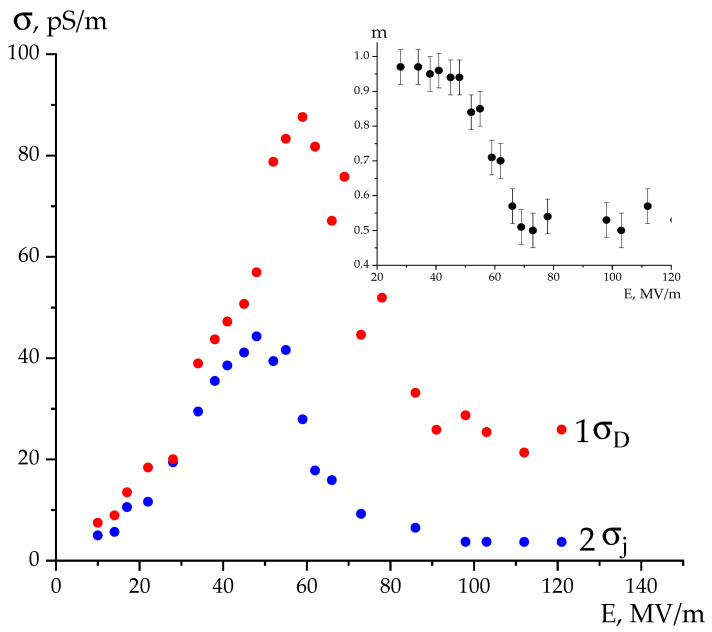
Conductivity field dependences obtained by different methods; inset represents the field dependence of the empirical parameter m in Equation (3).

One of the objectives of this paper was to compare the conductivity values calculated with both methods. Figure 4 shows that kinetic curves do not behave monotonously regarding the external field. This is shown further in Figure 5.

**Figure 6:**
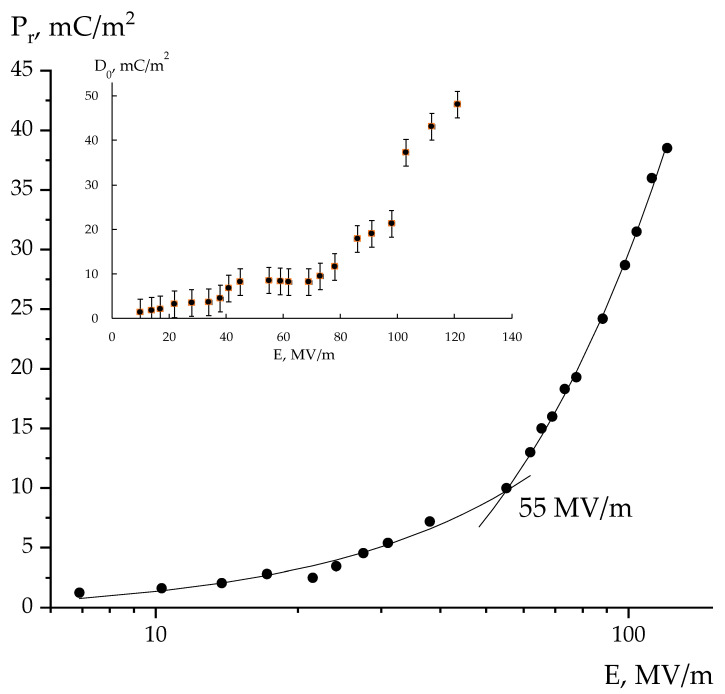
Field dependence of remnant polarization in the polarizable film; insertion is the *D*_0_ parameter field dependence characterizing the fast stage of the charge response.

It follows from Figure 5 that for all fields the values of *σ_D_* are greater than *σ*_j_. The authors conclude that the polarization response in Equation (3) is determined by both second and third summands. Figure 5 shows that both dependences have the form of curves with a maximum in the field region of 50–60 MV/m. Up to these fields, the conductivity increase qualitatively agrees with that forecasted by the Poole–Frenkel equation:

(5) $$j_{PF}=n_{T}\mu qEexp\left[-\frac{E_{T_{0}}-\beta_{PF}E^{\frac{1}{2}}}{kT}\right]$$

where the field reduces the value of potential barrier while transporting the *q* charges with the *n_T_* concentration and the *μ* drift mobility. As can be seen, at fields above 50–60 MV/m, this equation stops working. Figure 6 provides an indication of the possible reason for the noted “abnormal” behavior.

Allegedly, there are two areas of the curve behavior. The approximate field where these sections of the curve intersect is 55 MV/m. It is known that for the polymers under consideration, this figure corresponds to the *E_c_* coercive fields [1,2,3,16]. Thus, at fields above the *E_c_*, conductivity begins to “abnormally” decrease. Based on the one type of media, the following could be written:

*σ= nμq,* (6)

Considering the ferroelectrics, according to (6) above, the decrease in conductivity with an increasing field above *E_c_* could be most realistically associated with a decrease in the *n* concentration. Regarding the ferroelectrics above this field, the crystals’ polar planes begin to “rotate” in the external field direction. The marked polar planes are the deep traps for charges. Therefore, the effective carrier capture cross-section increases with the growing field. This should lead to a decrease in the *n* concentration and hence the conductivity, as can be seen in Figure 5. The insert to this figure also notes the decrease in the empirical coefficient m in Equation (3) with such fields. Thus, if up to *E_s_ m*~1, then it turns out to be ~0.5 above such a field.

As noted above, the bound charges (the third term in Equation (3)) will contribute to the kinetic curve *D-t*. This should also be observed at fields smaller than the *E_c_*. The non-linearity of the behavior of *D-t* in such fields (Figure 6) is an indirect sign.

**Figure 7:**
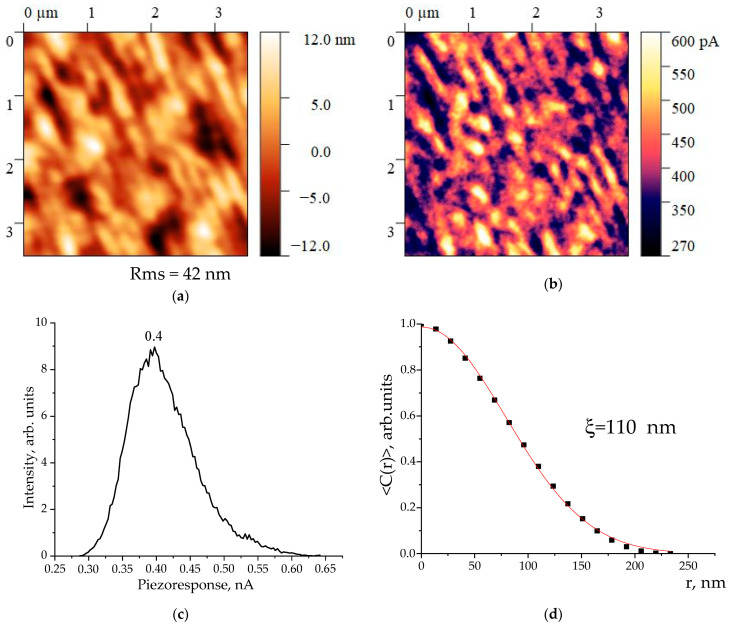
Copolymer film surface topography (**a**), piezoresponse pattern (**b**) with the average distribution curve (**c**,**d**) autocorrelation function.

In this regard, we review the mechanisms of the domain structure formation in the considered copolymer, as well as in the polymeric ferroelectrics of this class. It would be possible to introduce a simplified scheme according to which the domain is a polar crystal immersed in the amorphous matrix. In this case, the domain size can be estimated from Equation (1) using the X-ray diffraction data. The correctness of such an approach is defined by the fact that in crystallizing polymers, the width of the X-ray reflex is primarily determined, among the other factors, by the size of the coherent scattering region. Based on the data in Figure 3, the size in the direction of 110.200 (the most intense reflex) is calculated under Equation (1). It amounted to 8.9 nm. To confirm the proposed hypothesis, it is interesting to compare it with the domain size estimated from piezoelectric microscopy data. These data are presented in Figure 7.

The characteristic size of such domains *ξ* was found by calculating the charge distribution autocorrelation function depending on the *r* coordinate [18,19,20]:

*C(r)∝exp[−(r/ξ)^(−2h)],* (7)

where *h* is the exponent parameter (0 < *h* < 1) [18,19,20].

Figure 7d shows that the domain size is 110 nm. This is several times the size of the polar crystal (Figure 3). This leads to the conclusion that the domain providing a piezoresponse also includes the amorphous phase regions besides the crystal. This conclusion is non-trivial, but does not contradict the general conclusion that the mechanism of piezoelectricity in the polymers considered does not coincide with that in the inorganic ferroelectrics [1,2,3,13,14].

**Figure 8:**
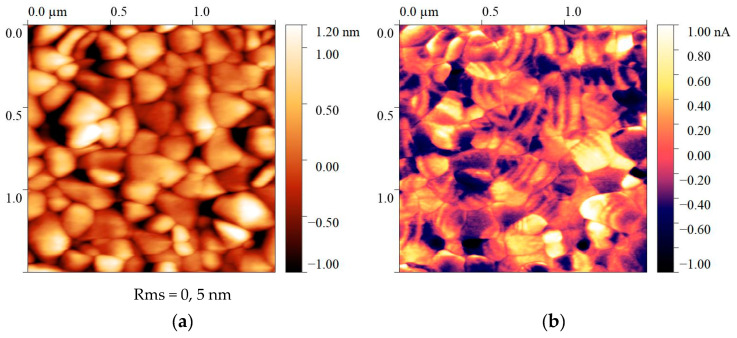
Surface topography of thin film PZT (**a**) and piezoresponse pattern (**b**).

Figure 8 represents the comparison of the surface topography of a 500 nm thick PZT film obtained by magnetron sputtering on a silicon substrate and the vertical piezoelectric response signal. The domain boundaries in inorganic ferroelectrics have a more pronounced contrast. Regarding polymers, the low contrast of the piezoresponse signals is for several reasons:

1. The present amorphous phase is in a liquid-like state, which implies that the chains of the amorphous phase are co-dynamical. The oscillating dipoles (electric field sources) will reduce the observed contrast.
2. Furthermore, the structure of the crystallizing polymers is not strictly biphasic, given the thermodynamic point of view, since the amorphous and crystalline phases do not have clear boundary interfaces. Here, one can observe tensegrity chains (constricting-tight chains) passing between them. This circumstance also does not contribute to high contrast of the piezoresponse signal.

Compared to inorganic materials, polymer ferroelectrics show a more complex two-phase structure. The features of such a structure affect the electrophysical properties of the material. For more clarity, the authors present a schematic model of the lamellar structure of VDF copolymers.

**Figure 9:**
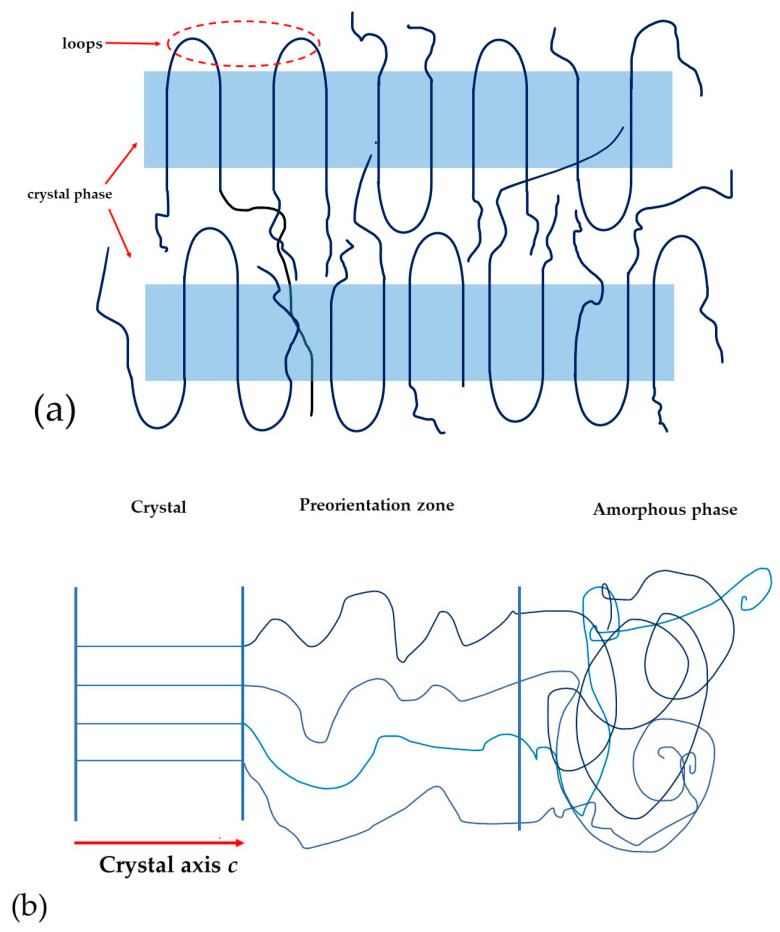
Sketch of the structure of a lamellar crystal of a ferroelectric polymer: (**a**)—crystals with three-dimensional ordering (color area) and possible defects (loops); (**b**)—schematic representation of the location of crystal, preorientation zone and amorphous phase.

Figure 9a shows a simplified model of the lamellar structure of the VDF copolymer. This figure shows that lamellar crystals comprise areas with their own three-dimensional ordering. Such areas are highlighted in blue color. Those areas with their own three-dimensional ordering structure contain the loops. The loop regions will not affect the crystal size calculated from the WAX data, but they can contribute to the ferroelectric domain size obtained from the PFM data.

In such systems, Figure 9b shows that it is possible to form a pre-orientation zone with an intermediate form of ordering. In this case, the size of the crystal is determined only by reflections from the crystal itself. Figure 9b shows the real system which also contains a preorientation zone. It will not contribute to the size of the crystal, but will affect the domain size.

Such features of the microstructure lead to the fact that the mechanism of piezoelectricity in the considered examples does not coincide with that in inorganic ferroelectrics [1,2,3,7,8]. According to the conclusions of these works, the main contribution to the piezoresponse mechanism is provided by electrostriction [21] and the so-called “size effect”, determined by high values of dielectric susceptibility and mechanical compliance due to the presence of an amorphous phase. The details of structural rearrangements in the amorphous phase depend on the understanding of its structural and dynamic properties for the crystalline polymers in general and for our materials in particular. General aspects of the structural details of the amorphous phase have already been noted by Flory, where it was stated that the transition from a crystal to the amorphous phase required the presence of the transition layer. A more general problem requires consideration of the structural details of the amorphous phase, mainly in crystalline polymers with flexible chains. In materials such as polyethylene and isotactic polypropylene, structuring processes in the marked phase up to formation of the mesomorphic state have been observed [22,23,24,25,26,27]. Considering polymeric ferroelectrics, which also belong to this class of polymers, the appearance of a fine structure in the amorphous phase is manifested in secondary crystallization [7,28]. Experimental evidence of such processes was obtained using DSC method, where, besides the main (high-temperature) melting process, the low-temperature heat absorption peak was also detected [12]. It could be assumed that peak halo with the angular position in the ~18° region (Figure 3) indicates formation of a fine structure in the amorphous phase of our copolymer.

It is possible to refer to certain details of the polarization process shown in Figure 4a. When a rectangular voltage pulse is applied, the response curve always includes two stages of the process: fast (*D*_0_) and subsequent slow, which in our case was tens of seconds. The used setup did not allow the kinetics of the fast charge response processes to be recorded. Emphasis was placed on analyzing the shape of the response curve, considering the slow processes. These include the bulk charge formation because of impurity and injected (at high fields) carriers should be taken into account. Due to their low mobility, this requires long periods, resulting in a partial reduction in the polar crystal depolarization field. The insert in Figure 6 shows a nonlinear increase *D*_0_ in the recorded value with the field. Such a curve should be analyzed considering the research data and relaxation processes in the considered polymers [12]. As noted, this phenomenon can be explained by cooperative rearrangements in the chains of the amorphous phase at higher fields. It follows that the mobility of the chains in the high frequency region (responsible for the fast *D*_0_ response) is associated with their co-motion in the amorphous phase.

## 4. Conclusions

The experimentally observed “abnormal” decrease in conductivity (for the one-dimensional case) at high fields (above *Ec*) in the ferroelectric copolymer of vinylidene fluoride with tetrafluoroethylene is associated with an increase in the carrier capture cross section. This is due to an increase in the concentration of deep traps as polar planes that “rotate” in the direction of the fields higher than the coercive ones. The unusual increase in high-frequency values of the loss factor after polarization is associated with conformational rearrangements of the amorphous phase chains, leading to an increase in the proportion of isomers in the planar zigzag conformation. At such field-induced transitions, the effective dipole moment of the kinetic units of the liquid-like amorphous phase is increased, leading to an increase in the dielectric loss factor measured in the experiment. High polarization fields (over two times higher than coercive fields) show the possibility of using such materials in capacitive energy storages and systems using the electrocaloric effect. Such systems make it possible to create, for example, solid-state refrigerators. Conformational changes in the chains of the amorphous phase were detected when the amplitude of the polarizing field increases. It is possible that the chains of the amorphous phase undergo additional crystallization. These data can optimize obtaining piezoelectric material based on copolymer of VDF with TFE. The identified feature of the amorphous phase involved in the polarization process should be studied to improve the polarization processes of films based on VDF.

## Data Availability

Data are contained within the article.
